# Enhancing the High-Solid Anaerobic Digestion of Horticultural Waste by Adding Surfactants

**DOI:** 10.3390/molecules29174061

**Published:** 2024-08-27

**Authors:** Wangliang Li, Zhikai Zhang, Shuzhen Mi, Shengyong Zhao

**Affiliations:** 1 Henan Academy of Sciences, Zhengzhou 450052, China; zhangzhikai@hgu.edu.cn; 2 CAS Key Laboratory of Green Process and Engineering, Institute of Process Engineering, Chinese Academy of Sciences, Beijing 100190, China; 3 University of Chinese Academy of Sciences, Beijing 100049, China; 4 School of Water Resources and Environment, Hebei GEO University, Shijiazhuang 050031, China; 5 Henan Vocational College of Water Conservancy and Environment, Zhengzhou 450008, China; mishuzhen1980@126.com

**Keywords:** non-ionic surfactant, horticultural waste, high-solid anaerobic digestion, kinetics model

## Abstract

The influence of adding surfactants on the performance of high-solid anaerobic digestion of horticultural waste was extensively investigated in batch systems. Adding Tween series and polyethylene glycol series non-ionic surfactants had positive effects on biogas production, resulting in 370.1 mL/g VS and 256.6 mL/g VS with Tween 60 and polyethylene glycol 300 at a surfactant-to-grass mass ratio of 0.20, while the biogas production of anaerobic digestion without surfactants was 107.54 mL/g VS. The optimal and economically feasible choice was adding Tween 20 at a ratio of 0.08 g/g grass in high-solid anaerobic digestion. A kinetics model reliably represented the relationship between surfactant concentration and biogas production. The mechanism of surfactants working on lignocellulose was investigated. The improvement in high-solid anaerobic digestion by adding surfactants was attributed to the interaction between lignocelluloses and surfactants and the extraction of biodegradable fractions from the porous structure. An economic analysis showed that adding Tween 20 was likely to make a profit and be more feasible than adding Tween 60 and polyethylene glycol 300. This study confirms the enhancement in biogas production from horticultural waste by adding non-ionic surfactants.

## 1. Introduction

At present, the tremendous increase in population size is leading to rapid energy and resource consumption and will generate 2.2 billion tons of municipal organic waste all over the world by 2025 [1]. Meanwhile, in China, the annual generation of municipal solid waste has reached a horrible amount, in which horticultural waste plays a big part. Disposing of biomass waste by anaerobic digestion (AD) is very promising [2,3,4,5,6] because AD has many advantages, such as producing energy-rich biogas, reducing biomass waste, killing pathogens, and producing little secondary pollution [7,8,9,10]. High-solid anaerobic processes are especially attractive because the quantity of water needed is substantially reduced, and the digester size is minimized [11,12]. Nevertheless, because of the high solid content, there are some limitations to the high-solid anaerobic digestion (HSAD) of horticultural waste, such as the serious concentration gradient in the raw materials, difficulties in mass transfer, the easy accumulation of inhibitory compounds, difficulties in controlling pH, etc. [13,14]. Moreover, horticultural waste is a complex biopolymer that is primarily composed of cellulose, hemicellulose, and lignin. The entangled biomass ultrastructure has inherent properties, such as strong lignin layers, low cellulose accessibility to chemicals, and high cellulose crystallinity, which inhibit biomass digestibility. This situation offers both challenges and promises for biomass biorefinery development to utilize cellulose from lignocellulosic biomass.

Non-ionic surfactants can be used to improve the hydrolysis of lignocellulosic waste because they can change the complex structure of horticultural waste and make the surface more accessible to enzymes [15]. The use of surfactants could reduce energy utilization in the hydrolysis process [16]. Moreover, non-ionic surfactants have relatively low toxicity. Non-ionic surfactants, such as Tween 80 (T80), have a positive influence on the wet AD of palm oil mill effluent and enhance biogas yield [17]. Adding T80 and polyethylene glycol (PEG) 6000, individually or in combination, substantially increases cumulative hydrogen production and hydrogen yield [18]. Tween 20 (T20) is considered to be capable of modifying lignin surface properties to change its hydrophobicity, hydrogen bonding ability, and surface charges [19].

Except for the improvement in mass transfer, the toxicity and biodegradability of surfactants have great impacts on microbial performances in terms of growth and activity [20,21]. The anaerobic biodegradability and inhibitory effects on the methane production of sodium lauryl sulfate, sodium dodecyl benzene sulfonate, and trialkyl-methyl ammonium chloride have been evaluated with two different kinds of anaerobic sludge. However, an inhibitory effect on methane production is observed in both sludges at 500 mg/L, which indicates that sodium dodecyl benzene sulfonate is not biodegradable under anoxic conditions [22]. The performances of cationic and anionic surfactants are not as good as those of non-ionic surfactants, which may be because the charges of the cationic or anionic surfactants cause the disturbance and deformation of the enzyme structure and then inhibit the AD process. Conversely, Tween 80 is reported to have no discernible impact on the microbial community [23]. Therefore, non-ionic surfactants are used in this experiment to study their ability to improve the HSAD process.

Although the effects of non-ionic surfactants have been studied by many researchers, most of their studies have focused on wet digestion processes, using substrates such as palm oil mill effluent [17,24], algal water [25], and so on. In this experiment, grass, a typical type of horticultural waste, is used as the substrate in the HSAD process. To the best of our knowledge, few studies have explored the effect of non-ionic surfactants on biogas production from solid waste via the HSAD process [26]. Additionally, the mechanism of interaction between surfactants and horticultural waste has not been previously considered. It is important to clarify the ability of surfactants to enhance biogas yield in this context.

The aim of this research was to develop a promising strategy for utilizing non-ionic surfactants to enhance biogas production through the HSAD of grass. To achieve this, the practice of adding different surfactants to horticultural waste was evaluated in laboratory batch tests. The mechanism of adding surfactants to enhance biogas yield was verified by biodegradability and Fourier transform infrared spectroscopy (FTIR). An economic assessment was performed by analyzing three different scenarios to implement an energy integration design, to study its economic feasibility, and to set the basis for a process scale-up.

## 2. Results and Discussion

### 2.1. Effect of Adding Surfactants on Biogas Production in High-Solid Anaerobic Digestion

#### 2.1.1. Adding Tween 20 in High-Solid Anaerobic Digestion

**Figure 1:**
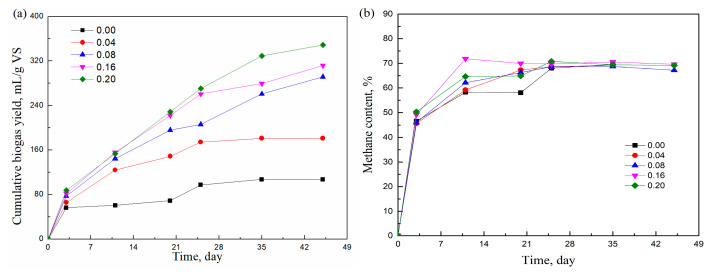
Effects of the T20/grass ratio (**a**) on cumulative biogas yield of grass and (**b**) on methane content.

Figure 1a shows the effect of adding T20 on cumulative biogas production. It can be seen that the cumulative biogas yields were increased by adding T20 surfactant. When the ratio of T20/grass was increased from 0.04 to 0.08, the cumulative biogas yield increased from 181.2 mL/g VS to 291.9 mL/g VS, while with a further increase in the ratio of T20/grass to 0.20, the cumulative biogas yield was augmented only by 57.2 mL/g VS compared with the yield at the ratio of 0.08. The biogas yield was obviously enhanced when T20 was added. However, the enhancement in HSAD became slight when the concentration of surfactants was above the ratio of 0.08. Adding T20 also displayed positive effects on the simultaneous saccharification and fermentation of steam-pretreated softwood [24]. Patel and Madamwar [25] observed a good result when adding T80 (300 mg/L), where the methane production increased by 48.5%. The improvement in HSAD by adding T80 was due to the formation of favorable active sites and the coupling of sequential reactions for the conversion of polymers into soluble substances, fatty acids, and, finally, into gases, which may also explain the enhancement in T20 on biogas production. Figure 1b shows the effect of adding T20 on the methane content. It can be seen that the methane concentration increased sharply in the first 3 days, then increased slowly to about 65%, and, finally, kept constant in the range of 65% to 70%.

Table 1 shows the properties of digestate after anaerobic digestion. In Table 1, it can be seen that all the samples showed a pH in the range of 7.33–7.69, which was suitable for methane production. The ammonia content increased with the increase in the mass ratio of T20 to grass, which meant the increase in surfactant concentration improved mass transfer by the extraction of biodegradable fraction from grass. The SCOD decreased from 613.9 mg/g TS to 5.3 mg/g TS, and VS removal augmented from 50.10% to 64.35% with the increased T20 addition. This behavior was consistent with that observed by Leaño and Babel [17], who found that the percentage of COD removal in palm oil mill effluent increased from 30% to 58% after the addition of T80. In addition, T80 was reported to promote VFA production by a 50% increase [26]. The enhancement effect of T80 may result from increasing the solubility of hydrophobic organic compounds because of the formation of micelles in the solution [27]. Thus, T80 helped to promote the transport of the biodegradable fractions to the surface of bacteria during the AD process, resulting in a faster degradation process. T20 possessed a similar structure and properties to T80; therefore, the mechanism of T20 in enhancing mass transfer was approximately explained.

#### 2.1.2. Adding Tween 60 in High-Solid Anaerobic Digestion

**Figure 2:**
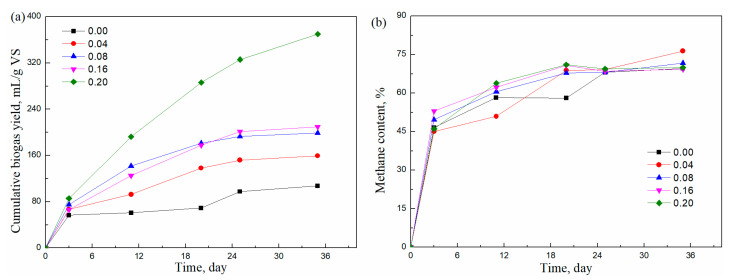
Effects of the T60/grass ratio (**a**) on the cumulative biogas yield of grass and (**b**) on the methane content.

Figure 2a shows the effect of T60 addition on the cumulative biogas production. It can be seen that the cumulative biogas yields increased when adding T60 surfactant. The biogas yield with a T60/grass ratio at 0.04 was enhanced by 48% in comparison with the Tween-free sample. After increasing the mass ratio of T60/grass from 0 to 0.2, the cumulative biogas yield substantially increased from 107.5 mL/g VS to 370.1 mL/g VS. The result of adding T60 followed the same trend as that of T20. Similar results were observed after adding T80 in biohydrogen from palm oil mill effluent, and when the T80 concentration increased from 0.05% to 0.5%, the hydrogen production increased from 33.1% to 77.0% compared with the control [17]. As we know, Tween is a series of esters formed by the reaction of sorbitol with different fatty acids—specifically, Tween 20 with lauric acid, Tween 60 with stearic acid, and Tween 80 with oleic acid—indicating that their physicochemical properties and effects are similar. In addition, there were also some differences between adding T60 and T20 in the anaerobic digestion of horticultural waste. The duration of HSAD when T60 was added was 35 days, 10 days shorter than that of HSAD when T20 was added. The biogas yield of grass with a T60 addition ratio of 0.20 was higher than that of grass with T20 addition at the same ratio, while the biogas yields with T60 addition were lower than those with T20 addition at the ratios of 0.04, 0.08, and 0.16. This revealed that T20 enhanced the biogas production of grass even at a relatively low dose, whereas T60 at a high concentration could obviously improve HSAD. This may be due to the different structure of the Tween product.

In Figure 2b, it can be seen that the methane concentration followed a similar trend as that shown in Figure 1b. The methane concentration increased sharply to around 50% within the first 3 days and then increased slowly to about 65%. Afterward, the methane concentration remained constant in the range of 65% to 75% until the end of digestion. Compared with adding T20, when adding T60, the methane concentration was slightly higher.

Table 2 shows the analysis of digestate when adding T60. It can be seen that the pH values of all samples were in the range of 7.56–7.76, which were suitable for anaerobic digestion and methane production. The concentration of SCOD decreased sharply with the increase in the mass ratio of T60 to grass. When the mass ratio of T60/grass was 0.20, the SCOD concentration was 0, which meant that the soluble chemicals were completely used for methane production. VS removal increased from 50.0% to 65.9% when the T60/grass ratio was raised from 0 to 0.20, further confirming the positive impact of adding surfactant on HSAD performance. At the end of digestion, half of the VS was consumed by microbes. However, when T60 was added to the substrate at the ratios of 0.04, 0.08, and 0.16, the VS removal increased slightly. In contrast, when T60 was added at the ratio of 0.20, 65.9% of the VS was digested during HSAD, indicating that T60 showed great improvement at a high concentration.

#### 2.1.3. Adding Polyethylene Glycol 300 in High-Solid Anaerobic Digestion

**Figure 3:**
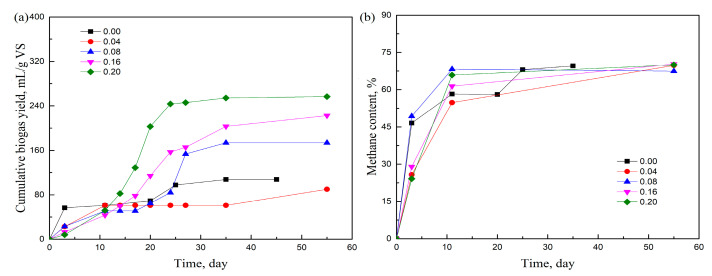
Effects of the PEG 300/grass ratio (**a**) on the cumulative biogas yield of grass and (**b**) on the methane content.

Figure 3a shows the effects of the PEG 300/grass ratio on the cumulative biogas yield of grass. It can be seen that the production of biogas was postponed when adding PEG 300 in comparison with the PEG-free sample. When increasing the dose of PEG 300, the biogas production was lower within the first five days, which indicated the negative effect of PEG on the initial stage of HSAD. Huang et al. [28] observed that the growth of anaerobic microbes on PEG 600 was delayed by about 1 day, and afterward, the biomass of microbes increased rapidly while PEG 600 decreased smoothly. This result is coincident with our research, in which the cumulative biogas yield increased after a few days. Although most researchers consider that PEG is harmless for living organisms [24], it appears that the positive or negative effects of surfactants on microbes vary depending on the microbial strain [29,30]. The results in Figure 3a suggest that PEG slightly hindered the hydrolysis stage to some extent. However, this impediment was not substantial, as the biogas yield increased significantly from the fifth day until the end of digestion when the PEG 300/grass mass ratio exceeded 0.08. The biogas yield reached 256.64 mL/g VS with a PEG/grass ratio of 0.20, which was about 1.4 times higher than the control (PEG/grass ratio of 0.00). When anaerobic digestion after adding PEG 300 was compared with that after adding T20 and T60, the duration of digestion was reduced from 35 days to 25 days, but the biogas yield was not that tremendously improved. Since hydrolysis is the first step in the AD process, this means that cellulose, as a biodegradable part of grass, was first degraded into small molecules such as sugar. PEG was also reported to have an adverse effect on sugar production compared with T80 and SDS when treating coconut husk in combination with surfactant-assisted subcritical water and enzymatic hydrolysis [31]. Fang et al. [32] also found that PEG did not improve the release of reducing sugars during the microwave-NaOH pretreatment of peanut shells. Therefore, the biogas production in HSAD with PEG was not as high as that in AD when T20 or T60 was added. The AD process was complex, and the impact of PEG on HSAD was influenced by multiple factors. Ultimately, while PEG showed an improvement in biogas production compared with the PEG-free sample, a slightly negative effect was observed at the initial stage. Figure 3b shows the effect of the PEG 300/grass ratio on methane concentration. The methane content increased quickly when the ratio of PEG 300/grass was 0.08 and 0.00. While at the ratio of 0.20, the methane content was the highest among all the samples. Despite the different concentrations of surfactants, the methane content of all the samples increased to 60–70% after 10 days.

### 2.2. Kinetic Analysis by Modified Gompertz Model

A modified Gompertz model was employed to simulate methane production during the HSAD process, and the principal kinetic parameters were investigated by regression analysis. The simulation results are summarized in Table 3. The simulated maximum cumulative methane yield (A) was similar to the experimental yield, indicating that the cumulative methane yield curve fitted well with the Gompertz model. The λ values representing lag phase time were almost zero when evaluating the effect of adding T20, T60, and the control, revealing that the hydrolysis stage proceeded smoothly for these samples. In contrast, when PEG 300 was added into HSAD, the λ values increased with the augmentation of the dose of PEG 300, which was coincident with the decrease in cumulative biogas production during the first five days and confirmed the negative effect of PEG 300 on the initial stage of HSAD. In contrast, Singh et al. [33] found that the lag phase of biohydrogen decreased from 7–12 h to 1–3 h with PEG addition. Because of the different substrates and disparate microbial processes, the effect of PEG could be inconsistent. The maximum methane production rates (U) were enhanced when adding T20, T60, and PEG 300 into HSAD compared with the control, indicating that adding these non-ionic surfactants accelerated the hydrolysis rate of the substrate and improved digestion. When T60 was added into the substrate, the U value was much higher at the T60/grass ratio of 0.20 than at the other three ratios, which supported the previous result that T60 at a high dose apparently improved HSAD, whereas T20 showed enhancement in HSAD even at a relatively low concentration. Moreover, the R^2^ obtained from the control was 0.807, which was much lower than those obtained from the samples with T20 or T60 added. This was probably attributed to the limitation of mass transfer in HSAD, causing the inferior fitting by the Gompertz model, while adding T20 or T60 obviously improved the HSAD process and showed the ideal growth curve of methane production. The R^2^ values of the samples after adding PEG 300 at the ratios of 0.04 and 0.08 were also poor because of the impeding effect of PEG 300 on the initial stage of AD, while the other two samples with PEG 300/grass ratios of 0.16 and 0.20 were 0.995 and 0.988, respectively. The effect of PEG 300 on HSAD was the balance of enhancement and hindrance, and the enhancement took the dominant place when the PEG 300/grass ratios were 0.16 or 0.20.

### 2.3. Biodegradability of Surfactants

**Figure 4:**
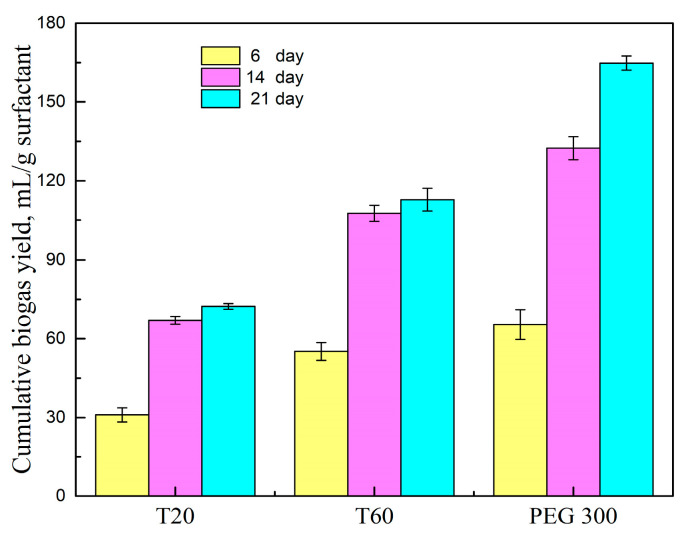
Cumulative biogas production generated by surfactants.

Since surfactants can be digested by anaerobic microbes, the biodegradability of T20, T60, and PEG 300 was evaluated by biogas yield [22]. As shown in Figure 4, the cumulative biogas yields of T20 and T60 changed little after 14 days, while the biogas yield of the samples after adding PEG 300 kept increasing along with the test. The maximum biogas yield was achieved by PEG 300, at 164.8 mL/g surfactant, which was 2.3 times the biogas yield of T20 and 46% higher than that of T60. It was notable that PEG 300 was easily biodegradable because anaerobic microbes can use PEG as a sole carbon source [28]. The biodegradability of T20 and T60 was much lower than PEG 300 because non-ionic surfactants are limited in biodegradation processes; only water-soluble molecules can be metabolized by microorganisms [34]. During the enhanced anaerobic digestion after adding surfactants, the addition ratio of surfactants to grass did not exceed 0.2, indicating that the increased biogas production from surfactants was lower than 32.9 mL/g when adding PEG 300, 22.5 mL/g when adding T60, 14.5 mL/g when adding T20. But the biogas yield of samples with surfactant addition increased much more than the biogas yield from surfactant degradation, suggesting that the enhancement in biogas production was due to the promotion of surfactants on HSAD, and the contribution of surfactant degradation could be ignored.

### 2.4. Improvement in the Mechanism of High-Solid Anaerobic Digestion by Adding Surfactants

**Figure 5:**
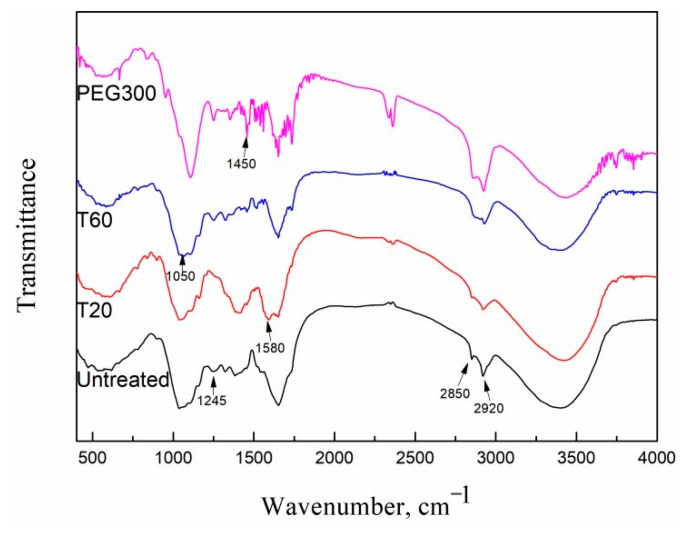
Effect of adding surfactants on the structure of grass.

The substrate’s structure was analyzed using FTIR to gather additional evidence supporting the enhancement of HSAD through the addition of surfactants. Therefore, the samples with the highest biogas production (i.e., all substrates mixed with PEG 300, T60, or T20 at the ratio of 0.20) were chosen to compare with the untreated grass. Here, it should be clear that the samples analyzed were mixed well with surfactants before anaerobic digestion. Figure 5 shows the FTIR spectra reflecting the structural variation in the functional group. There was an obvious broad absorption band at 3250~3500 cm^−1^. Some researchers ascribed the broad peak to the stretching vibration of the –OH group [35], whereas it was better to consider the peak as the stretching vibration of the –OH group extended by hydrogen bonding between molecules. Because hydrogen bonding extensively existed among lignin, hemicelluloses, and cellulose, the hydrogen bonding showed a broad absorption band while the stretching vibration of the –OH group was a relatively sharp peak. Therefore, the peak in the range of 3250~3500 cm^−1^ was the result of the stretching vibration of the –OH group and hydrogen bonding. The intensity of this peak slightly decreased after T20 and T60 were added into the substrate, indicating the intrusion of Tween into the lignocellulose structure, causing the weakness of hydrogen bonding, which further improved the separation of cellulose from lignin and provided more accessibility for the microbes. This may be part of the reason for the enhancement caused by T20 and T60 in the hydrolysis process in HSAD. Nevertheless, the intensity of this peak was strong when the substrate was added with PEG 300. It was reported that the hydrophobic parts of lignin such as phenyl and –CH_2_ and –CH_3_ groups will interact with the –CH_2_ groups in PEG, and there were hydrogen bonds between phenolic hydroxyls and the ether oxygens in PEG [36]. These interactions and hydrogen bonding reinforced the absorption peak in the range of 3250~3500 cm^−1^, indicating the bonding of PEG 300 to lignin. The consolidated lignocellulose structure may have impeded the access of anaerobic microbes and thus delayed the hydrolysis process, causing the hindrance of PEG 300 in the initial stage of HSAD.

The absorption peaks at 2850 cm^−1^ and 2920 cm^−1^ predominantly arose from symmetric and asymmetric stretching of –CH bonds [37,38]. The absorption became stronger when PEG 300 was added compared with the untreated grass and those added with Tween, which provided further proof of the interaction between lignocelluloses and PEG 300. The peaks at 1450 cm^−1^ and 1580 cm^−1^ were attributed to phenyl ring skeletal vibrations of lignin macro molecules [39]. The small peak that appeared at 1245 cm^−1^ was due to the in-plane bending vibration of –OH, probably in cellulose. The absorption band at 1050 cm^−1^ was the stretching vibration of C-O and C-O-C, indicating the existence of pyran ring skeleton vibration. The peaks in the range of 1050~1580 cm^−1^ exhibited great differences when the substrate was added with PEG 300 in contrast to the untreated grass and those added with Tween. The differences confirmed the interaction between the hydrophobic parts of lignin and PEG [36].

**Figure 6:**
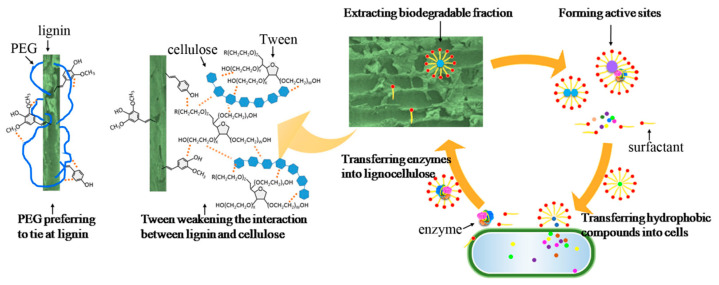
The mechanism in which surfactants promote mass transfer in HSAD (the images of lignin and cellulose in the composition of the substrate are schematic).

The FTIR gave deep insight into the interaction between the substrate and surfactants in the chemical structure aspect and made it much easier to explain the effect of surfactants such as T20, T60, and PEG 300 on the substrate. Based on the biogas production, properties of digestate, kinetic analysis, and IR spectrum during HSAD, the mechanism of HSAD improvement by adding surfactants became clear, which is illustrated in Figure 6. 

First, the interactions between the surfactants and lignin are somewhat different for PEG 300 and Tween. Some PEG 300 is tied to lignin owing to the interactions between the -CH_2_ groups in PEG and the hydrophobic parts of lignin such as phenyl and the –CH_2_ and –CH_3_ groups [36], which was proven by the FTIR spectrum. Contrary to PEG, Tween could promote the hydrolysis stage by modifying lignin surface properties, such as changing its hydrophobicity, hydrogen bonding ability, and surface charges [19]; thus, the biodegradable fractions are easier to remove from the cages of lignocellulose, which was verified by the high ammonia nitrogen concentration in the digestate. Although PEG and Tween both contain the –(CH_2_CH_2_O)– group, the reason for the combination of lignin with PEG instead of with Tween probably is the steric hindrance that prohibits Tween from adsorbing onto lignin. As Figure 6 shows, Tween intervenes in the middle of lignin and cellulose and weakens the interaction, especially the hydrogen bond between them, while PEG goes deep into the lignin structure and ties close to it. This consolidated structure likely hinders microbial access, resulting in the prolonged lag phase observed during digestion with PEG 300 addition. In contrast, the addition of Tween releases more biodegradable fractions, accelerating biogas production in the initial stage of HSAD.

Despite the hindrance caused by PEG 300 in the initial stage of HSAD, the overall biogas production was significantly increased by adding surfactants like PEG 300, T20, and T60. This is because these surfactants can easily penetrate the interior of lignocellulose, where cellulose is closely bound to hemicellulose and lignin. Surfactants help to extract the hydrophobic part from lignocelluloses by forming emulsions so that the biodegradable fraction is enhanced and becomes available to microbes [29,40]. Another effect of surfactants is that the hydrophobic molecules are surrounded by surfactants in the center of the micelle, which provides active sites for the reaction between the hydrophobic molecules and enzymes [41]. In addition, adding surfactants reduces the surface tension and viscosity of the substrate [42]; consequently, hydrophobic compounds move easily into microbe cells, and enzymes transfer well from cells to the solid substrate or liquid media.

### 2.5. Economic Assessment

An economic analysis was carried out to investigate the feasibility of adding surfactants such as Tween 20, Tween 60, and PEG 300 to improve HSAD performance. The analyzed scenarios adopted the ratio of surfactants/grass of 0.08, 0.16, and 0.20 for T20 and 0.20 for both T60 and PEG 300, at which the methane production was above 140 mL/g TS. The methane yield of grass without adding surfactant was 51.9 mL/g TS. Therefore, the extra methane produced per gram of grass after adding surfactants could be calculated. Assuming all the methane could be converted to electricity without heat loss, the profit was obtained by multiplying the methane yield by its calorific value and electricity price. The price of the additional methane was calculated based on previous reports, which indicate that the calorific value of methane is 11 kWh/Nm³ and the price of electrical energy is 0.15 EUR/kWh [43]. The price of surfactant was obtained by interviewing a local company. Thus, the ratio of cost to profit was obtained, as shown in Table 4. It is important to note that the ratio was merely a simplified index of economic analysis, omitting many real conditions such as fiscal subsidy, maintenance, depreciation of devices, transportation, construction cost, and so on.

As Table 4 shows, the ${Ratio}_{cost}$ of the scenario with T20 at 0.08 was 0.85, which was lower than 1.00, indicating that the practice of adding T20 at the ratio of 0.08 had the potential to make a profit. The other scenarios were above 1.00, resulting from the high adding ratio of surfactants, indicating the cost of chemical reagents was much higher than the profit generated by the increased biogas production. Based on these results, adding T60 or PEG 300 into HSAD was not economically feasible. However, it was still meaningful to add these surfactants to enhance HSAD, which provided an option for the exploration of improving mass transfer in HSAD. It also pointed toward a direction to find more effective and cheap surfactants to enhance HSAD.

## 3. Materials and Methods

### 3.1. Feedstock and Inoculum

Grass was collected from the yard of the Institute of Process Engineering. The grass was shredded and homogenized into small pieces (approximately 2 cm in length for grass). The anaerobic sludge was obtained from a Beijing sewage treatment plant and had a pH of 7.62. The sludge was centrifuged at a rate of 5000 rpm for 5 min, and the sludge cake was used as inoculum for high-solid digestion. The characteristics of the grass and inoculum sludge are presented in Table 5.

### 3.2. Surfactants

Tween 20 (T20) and Tween 60 (T60) are esters formed by different fatty acids with sorbitol. Tween 20 is laurate, while Tween 60 is stearate. Both of them are non-ionic surfactants and viscous, water-soluble yellow liquids often used in food and other products as emulsifiers. Polyethylene glycol (PEG) is a polyether compound that has several advantageous chemical properties, such as being typically biologically inert, non-immunogenic hydrophilic, and highly flexible, which make it especially useful in various biological activities. The physical characteristics of T20, T60, and PEG 300 are presented in Table 6.

### 3.3. High-Solid Digestion

The HSAD experiment was carried out in a 250 mL flask containing a substrate of 5.00 g VS at 35 °C. The initial TS of the HSAD was controlled at 20%. After inoculation, all batch reactors were purged using nitrogen gas to create an anaerobic condition. During the digestion process, biogas sampling was taken regularly using Tedlar bags together with pH and other analytic measurements.

The batches were incubated at surfactant/grass ratios of 0, 0.04, 0.08, 0.16, and 0.20 with Tween 20, Tween 60, and PEG 300 as surfactants. After the surfactants were added to the batches, the flasks were incubated at 35 °C for 45 days. The grass had a low buffering capacity, which made it more sensitive to a fast drop in pH under dry anaerobic digestion because of the generation of volatile fatty acids. Therefore, the initial pH of the feedstock was adjusted to 7.0 using sodium bicarbonate.

### 3.4. Kinetic Study

The modified Gompertz equation [44,45] was used to describe the biogas potential and the maximum biogas production rate at different dosages of T20, T60, and PEG 300.

Y = A × exp{−exp[Ue/A (λ − t) + 1]}(1)

where

Y—Cumulative methane production, N mL/g VS at any digestion time t;
A—Methane yield potential, N mL/g VS;
U—Maximum rate of methane production, N mL/(gVS·d);
λ—Lag phase period to produce methane, days;
t—Digestion time at which cumulative methane production Y is measured, d;
e—Mathematical constant (2.718282).

The kinetic parameters of A, U, and λ were simulated for each batch reactor using non-linear regression with the help of Origin software.

### 3.5. Analysis Methods

The grass samples were analyzed for TS and VS contents according to the standard methods of the American Public Health Association [46]. Rapid simultaneous determination of the carbon, hydrogen, nitrogen, and sulfur (CHNS) contents in the grass and anaerobic sludge was carried out in the Elemental Analysis Laboratory with the Elementar Vario Micro Cube instrument (Elementar, Hanau Germany). The cellulose, hemicellulose, lignin contents, NDS, and Ash were analyzed according to the procedure of Van Soest [47]. Soluble COD (SCOD) and the ammonia content were analyzed using the method described by Fabio Kaczala [48]. The daily biogas production was recorded by the water displacement method, and the cumulative biogas volume and methane yield were calculated after correction at standard temperature and pressure. Methane and carbon dioxide in the biogas were measured by gas chromatography (PerkinElmer, Waltham, MA, USA) equipped with a 2 m × 3 mm stainless steel column packed with TDX-01 and a thermal conductivity detector. The temperatures of the detector, injector, and oven were 150, 150, and 120 °C, respectively. Infrared spectroscopy was performed using an FTIR spectrophotometer (PerkinElmer, Waltham, MA, USA). The samples were mixed with KBr powder in a ratio of 1:100 in mg to prepare the KBr pellets for analysis.

### 3.6. Economic Analysis

An economic analysis was carried out to investigate the feasibility of adding surfactants such as Tween 20, Tween 60, and PEG 300 in high-solid anaerobic digestion. Numerous studies in the literature have conducted economic analyses that consider factors such as staff costs [49], heat loss [50], construction costs [18], and revenues from biogas, fertilizers, and environmental benefits [51]. However, these economic analyses adopted the ideal situation and made some assumptions by omitting some conditions, for example, intermission caused by maintenance, depreciation of devices, transportation, energy conversion efficiency, and so on, which should have been covered in the cost. One or two papers could not complete a sufficient feasibility study for anaerobic digestion because there were many factors to be considered. Therefore, to simplify the economic analysis and gain a preliminary economic estimation of HSAD as a reference, an index was introduced, as shown in equation 2. It was defined as the ratio of the cost of strategies input to the price of extra methane [52].

Ratio_cost_ = Cost of surfactants input/Profit of extra methane (2)

where extra methane is the methane production after adding surfactants minus the one without surfactants.

## 4. Conclusions

Adding surfactants can enhance anaerobic digestion and increase biogas yield. Increasing the surfactant-to-grass mass ratio improved both biogas yield and volatile solid (VS) removal efficiency. Among the surfactants tested, Tween 20 was identified as the optimal choice, with a suitable surfactant-to-grass mass ratio of 0.08. The kinetic study effectively modeled the biogas production process and explained the differences in performance among T20, T60, and PEG 300. The mechanism of surfactant enhancement was elucidated and well-supported by the FTIR spectrum. Unlike T20, which promoted the hydrolysis stage of anaerobic digestion, PEG 300 exhibited inhibitory effects during the initial stage because of its interaction with lignin. The economic analysis revealed that using surfactants like T20 to enhance HSAD was feasible and had the potential to be profitable in industrial applications.

## Figures and Tables

**Table 1:** Analysis of solid digestate when adding Tween 20.

T20/Grass	pH	Ammonia, mg/g TS	SCOD, mg/g TS	VS, %	VS Removal, %
0.00	7.68 ± 0.08	4.52 ± 0.16	613.9 ± 3.00	9.99 ± 0.01	50.0
0.04	7.33 ± 0.09	4.49 ± 0.08	321.6 ± 4.66	8.29 ± 0.05	58.55
0.08	7.65 ± 0.04	5.92 ± 0.14	48.3 ± 2.13	7.83 ± 0.09	60.85
0.16	7.69 ± 0.10	5.72 ± 0.05	19.4 ± 8.96	7.53 ± 0.04	62.35
0.20	7.54 ± 0.20	5.91 ± 0.17	5.3 ± 7.11	7.13 ± 0.06	64.35

**Table 2:** Analysis of solid digestate when adding Tween 60.

T60/Grass	pH	Ammonia, mg/g TS	SCOD, mg/g TS	VS, %	VS Removal, %
0	7.68 ± 0.08	4.52 ± 0.16	613.9 ± 3.00	9.99 ± 0.01	50.0
0.04	7.57 ± 0.03	4.85 ± 0.09	485.1 ± 6.06	9.76 ± 0.09	51.2
0.08	7.7 ± 0.11	6.39 ± 0.15	85.8 ± 9.82	9.56 ± 0.04	52.2
0.16	7.63 ± 0.06	6.72 ± 0.12	43.9 ± 5.79	9.48 ± 0.08	52.6
0.20	7.56 ± 0.02	7.43 ± 0.10	0 ± 2.61	6.83 ± 0.11	65.9

**Table 3:** Effect of different surfactant concentrations on the methane potential and maximum methane production rate.

Batches	A (N mL/g VS)	λ (d)	U (N mL/(gVS·d))	R^2^
Control (0%)	64.0 ± 10.8	0.0 ± 0.0	2.25 ± 0.5	0.807
Effect of T20				
0.04	102.7 ± 6.1	0.0 ± 1.8	5.63 ± 1.2	0.956
0.08	181.7 ± 19.7	0.0 ± 2.7	5.68 ± 1.1	0.952
0.16	200.5 ± 12.2	0.0 ± 1.7	7.64 ± 1.1	0.977
0.20	234.5 ± 18.2	0.0 ± 1.9	7.17 ± 0.9	0.978
Effect of T60				
0.04	94.0 ± 12.6	0.0 ± 2.8	4.27 ± 1.2	0.920
0.08	115.9 ± 8.6	0.0 ± 1.8	7.07 ± 1.7	0.954
0.16	133.9 ± 10.5	0.0 ± 1.7	6.65 ± 1.3	0.967
0.20	243.9 ± 15.6	0.5 ± 1.2	9.92 ± 1.1	0.988
Effect of PEG 300				
0.04	32.4 ± 4.0	-0.2 ± 3.9	2.08 ± 0.9	0.715
0.08	102.7 ± 19.2	5.2 ± 4.7	3.17 ± 1.1	0.816
0.16	146.5 ± 3.9	8.8 ± 0.6	6.32 ± 0.4	0.995
0.20	176.1 ± 5.5	10.0 ± 0.7	13.9 ± 1.5	0.988

**Table 4:** The ratio of cost consumption by adding a surfactant to the price produced by extra methane.

		T20	T20	T20	T60	PEG300
Surfactant per gram of grass	g/g TS	0.08	0.16	0.20	0.20	0.20
Methane per gram of grass	mL/g TS	153.2 ± 6.8	175.4 ± 7.3	190.1 ± 2.6	201.7 ± 4.7	149.3 ± 5.5
Extra methane per gram of grass	mL/g TS	101.3	123.5	138.2	149.8	97.4
Profit of extra methane	EUR/Kg TS	0.17	0.20	0.23	0.25	0.16
Price of surfactant	EUR/kg	1.77 ± 0.36	1.77 ± 0.36	1.77 ± 0.36	1.79 ± 0.31	1.39 ± 0.25
Cost of surfactant input	EUR	0.14	0.28	0.35	0.36	0.28
Ratio_cost_		0.85	1.39	1.55	1.45	1.73

**Table 5:** Characteristics of the grass and inoculum sludge used in this study.

Feed	Grass	Anaerobic Sludge
C, wt%	41.08 ± 0.70	43.96 ± 2.08
N, wt%	1.02 ± 0.02	2.40 ± 0.05
C/N ratio	40.27	18.32
S, wt%	<0.50 ± 0.04	0.67 ± 0.06
Total solid (TS),%	80.58 ± 0.98	21.25 ± 1.14
Volatile solid (VS),% Composition	69.81 ± 0.07	18.97 ± 0.05
NDS, %	16.68 ± 1.58	32.67 ± 1.77
Cellulose, %	42.49 ± 1.45	24.11 ± 1.15
Hemicelluloses, %	27.14 ± 1.37	19.52 ± 2.04
Lignin, %	12.67 ± 2.67	15.37 ± 2.85
Ash, %	0.67 ± 0.55	7.96 ± 0.71

Note: =TS was based on the total weight, while the others were based on TS. NDS is short for neutral detergent solutes.

**Table 6:** Characteristics of T20, T60, and PEG 300.

Parameter	Tween 20	Tween 60	PEG 300
Molecular formula	C_58_H_113_O_26_	C_64_H_126_O_26_	HO(CH_2_CH_2_O)_n_H
Shape	Yellow viscous liquid	Yellow or orange oily liquid or semi-gel	Colorless transparent liquid
Molar weight (g/mol)	1226.48	1311.68	~300
Density(g/mL)	1.095 g/mL at 25 °C	1.044 g/mL at 25 °C	-

## Data Availability

The data presented in this study are available on request from the corresponding author because of intellectual property rights.

## Abbreviations

anaerobic digestion	AD
Fourier transform infrared spectroscopy	FTIR
high-solid anaerobic digestion	HSAD
polyethylene glycol	PEG
Tween 20	T20
Tween 60	T60
Tween 80	T80
carbon	C
nitrogen	N
carbon-to-nitrogen ratio	C/N ratio
sulfur	S
total solid	TS
volatile solid	VS
neutral detergent solute	NDS
cumulative methane production at any digestion time	Y
methane yield potential	A
maximum rate of methane production	U
lag phase period to produce methane	λ
digestion time	t
mathematical constant	e
